# Anti-thyroid Peroxidase Antibody Positivity and Chronic Hyperglycemia as Key Risk Factors for Peripheral Artery Disease in Type 2 Diabetes: A Retrospective Cohort Study

**DOI:** 10.7759/cureus.86713

**Published:** 2025-06-25

**Authors:** N M Motachim Mahmud, Dale K Mwangi, Rafiul Islam Shuvo, Thomas Juby, Dhierin Jagdewsing, Wally Elijah, Mohamed Shuraim S Naushad, Fariya Iqbal, Akanksha Choudhary, Xiaochen Ji

**Affiliations:** 1 Department of Endocrinology and Metabolism, Second Affiliated Hospital, Dalian Medical University, Dalian, CHN; 2 Department of Clinical Medicine, Dalian Medical University, Dalian, CHN; 3 Department of Internal Medicine, Bangladesh Medical University, Dhaka, BGD; 4 Department of General Surgery, First Affiliated Hospital, Dalian Medical University, Dalian, CHN; 5 Department of General Surgery, Second Affiliated Hospital, Dalian Medical University, Dalian, CHN; 6 Department of Anesthesiology, Sir Run Run Hospital of Nanjing Medical University, Nanjing, CHN; 7 Department of Internal Medicine, Dalian Medical University, Dalian, CHN; 8 Department of Internal Medicine, Nanjing Medical University, Nanjing , CHN

**Keywords:** anti-thyroid peroxidase, chronic hyperglycemia, diabetes mellitus type 2, peripheral artery disease, thyroid autoantibody

## Abstract

Background: Anti-thyroid peroxidase antibody (TPOAb) affects metabolic and thyroid function parameters, which may worsen macrovascular complications. This study aims to find the association between chronic hyperglycemia, thyroid autoantibody, and peripheral artery disease (PAD) in type 2 diabetes mellitus (T2DM) patients, as well as examine important clinical demographic and metabolic variables.

Methods: This is a retrospective cohort study. Data from 2017-2023 of T2DM patients were collected. A total of 300 patients were included. Further, the T2DM patients are subdivided by TPOAb values. TPOAb categorized (0-60 IU/mL) as negative; TPOAb titer (>60 IU/mL) as positive. To analyze the data, IBM SPSS Statistics for Windows, Version 29.0.2.0 (Released 2022; IBM Corp., Armonk, New York, United States) was utilized. For continuous variables, the independent samples t-test for normally distributed data and the Mann-Whitney U test for non-normally distributed data were performed. The chi-square test was used to assess the association between categorical variables. The risk of PAD was assessed using univariate and multivariate binary logistic regression analysis. P<0.05 was set as statistically significant.

Results: The prevalence of PAD was significantly higher in the TPOAb-positive group (38.5%) compared to the TPOAb-negative group (21.6%, p = 0.004). Multivariate analysis finds that poorly controlled HbA1c had an increased risk of PAD compared to well-controlled individuals (OR: 2.14, p = 0.046). Those who had diabetes for >10-15 years showed the highest risk (OR: 3.67, P = 0.002), followed by those with diabetes for more than 15 years (OR: 2.22, P = 0.038). Similarly, TPOAb positivity was independently correlated with increased odds of PAD (OR: 2.48, P = 0.003) and therefore potentially heightened the risk of macrovascular complications in association with thyroid autoimmunity.

Conclusion: TPOAb positivity is associated with an increased risk of PAD. In addition, poor glycemic control and longer diabetes duration were associated with PAD.

## Introduction

Aberrations in immune regulation may trigger thyroid autoimmunity, in which the body generates thyroid-specific autoantibodies, such as anti-thyroid peroxidase antibodies (TPOAb) or thyroglobulin antibodies (TGAb), that initiate a targeted immune response against thyroid cells [1]. High levels of either TPOAb or TGAb serve as a clinical marker for the detection of autoimmune thyroid disease (AITD) [2]. Furthermore, type 2 diabetes mellitus (T2DM) patients have also been shown to have higher TPOAb presence, representing thyroid autoimmunity, which is also associated with metabolic disturbances in the patients. Elevated levels of TPOAb have been associated with adverse lipid and glycemic control, which are adverse risk factors for the progression of vascular complications [3]. T2DM is associated with hyperuricemia, which is defined as elevated serum uric acid levels. The prevalence of hyperuricemia in T2DM patients is reported from 10.7% to 45% [4]. Elevated serum uric acid levels have been associated with increased cardiovascular disease risk and factors such as elevated systolic blood pressure and higher body mass index (BMI) [5]. Peripheral artery disease (PAD) is a systemic atherosclerosis that manifests as constriction of peripheral arteries and reduced blood flow, especially to the limbs. Recent studies show that serum acid is positively associated with PAD, especially in male hypertensive patients [6]. Due to these interconnections, it is possible to hypothesize a triangular association of hyperuricemia, TPOAb positivity, and PAD in T2DM individuals. The exploration of this relationship is also important since it might provide some information on the complex interplay of metabolic and autoimmune factors for developing vascular complications in diabetes patients and to improve management strategies in this high-priority population.

## Materials and methods

The data used in the study were collected from the Second Affiliated Hospital of Dalian Medical University, China. Data from 2017 to 2023 were collected, and a total of 300 T2DM patients were included, which were subdivided by TPOAb levels. A post-hoc power analysis based on the observed PAD prevalence in TPOAb-positive (38.5%) and TPOAb-negative (21.6%) groups demonstrated that the study had >99% power (Cohen’s h = 0.37, α = 0.05, n = 300) to detect a statistically significant difference. This confirms the adequacy of the sample size for logistic regression. TPOAb titers were measured by the LSI Medience Corporation, Japan, using standard procedures, and the normal range (negative) was 0-60 IU/mL; a TPOAb titer >60 IU/mL was defined as TPOAb-positive. HbA1c was categorized into well-controlled (HbA1c <7%), moderately controlled (7% ≤ HbA1c <8%), and poorly controlled (HbA1c ≥ 8%) [7]. Persistent elevation of office systolic BP ≥140 and/or diastolic BP ≥90 mmHg, or 24-hr ambulatory blood pressure monitoring (ABPM) average ≥130/80 mmHg, defines hypertension [8]. This study design enabled an in-depth investigation of the long-term association between T2DM, thyroid autoantibodies, hyperuricemia, and peripheral vascular disease.

Patient selection

Individuals, who ranged in age from 25 to 90, had laboratory-verified thyroid hormone abnormalities or PAD in addition to proven T2DM were included in the study. Additionally, patients with malignant tumors, infectious diseases, a history of smoking or drinking alcohol during the last 10 years, pregnancy, mental health disorders, other thyroid conditions (such as Graves’ disease or thyroid cancer), or type 1 diabetes mellitus were not included in the study.

Data collection

The main independent variables were classified as either positive or negative TPOAb levels. The key dependent variable was whether PAD was present. PAD was defined based on established clinical criteria. Patients were considered to have PAD if they had a history of acute limb ischemia, critical limb ischemia, intermittent claudication, or Buerger’s disease or if they had an ankle-brachial index (ABI) <0.90 in either leg, as recommended by current guidelines. Additionally, age, gender, BMI, duration of T2DM, systolic and diastolic blood pressure, thyroid profile, lipid profile, fasting blood glucose, HbA1c, and length of hospitalization were examined.

Data analysis

To analyze the data, IBM SPSS Statistics for Windows, Version 29.0.2.0 (Released 2022; IBM Corp., Armonk, New York, United States), was utilized. For continuous variables, the independent samples t-test for normally distributed data (free triiodothyronine (FT3)) and the Mann-Whitney U test for non-normally distributed data (free thyroxine (FT4), low-density lipoprotein cholesterol (LDL-C), high-density lipoprotein cholesterol (HDL-C), total cholesterol (TC), triglycerides (TG), and glucose) were performed. The chi-square test was used to assess the significance of the association between categorical variables. The risk of PAD was assessed using univariate and multivariate binary logistic regression analysis. At p <0.05, statistical significance was established.

## Results

Table 1 compares the demographic and clinical features between TPOAb-negative (n = 222) and TPOAb-positive (n = 78) groups. Age distribution between the groups was not significantly different (p = 0.250), with similar proportions in the <45 years, 45-64 years, and ≥65 years categories. Sex distribution, though, was severely different (p = 0.008), as females predominated in the TPOAb-positive group 60 (76.9%) vs. 134 (60.4%). The groups exhibited similar diabetes duration (p = 0.338), with a slightly higher proportion of TPOAb-positive patients in the ≤5 years category (28 patients, 35.9%) and a reduced percentage in the >15 years category (14 patients, 17.9%). Also, BMI distribution was not significantly different (p = 0.342), with 86 patients (38.7%) vs. 35 patients (44.9%) in the <25 categories and 136 patients (61.3%) vs. 43 patients (55.1%) in the ≥25 categories. Blood pressure was also not significant (p = 0.094), with 104 patients (46.8%) vs. 28 patients (35.9%) in the non-hypertensive category and 118 patients (53.2%) vs. 50 patients (64.1%) in the hypertensive category. Nevertheless, PAD prevalence was significantly higher in the TPOAb-positive group of 30 patients (38.5%) vs. 48 patients (21.6%), p = 0.004, which might imply a possible connection between thyroid autoimmune disease and peripheral arterial disease.

**Table 1:** Comparing the clinical and demographic features of the TPOAb-positive and TPOAb-negative groups (N = 300) T2DM: type 2 diabetes mellitus; BMI: body mass index; PAD: peripheral artery disease; TPOAb: anti-thyroid peroxidase antibody

Variable	TPOAb -ve (n = 222)	TPOAb +ve (n = 78)	P-value
Age (years), n (%)			0.250
<45	15 (6.8%)	8 (10.3%)	
45-64	105 (47.3%)	29 (37.2%)	
≥65	102 (45.9%)	41 (52.6%)	
Sex, n (%)			0.008
Male	88 (39.6%)	18 (23.1%)	
Female	134 (60.4%)	60 (76.9%)	
Duration of T2DM, n (%)			0.338
T2DM ≤5 years	67 (30.2%)	28 (35.9%)	
T2DM >5–10 years	60 (27.0%)	24 (30.8%)	
T2DM >10–15 years	32 (14.4%)	12 (15.4%)	
T2DM >15 years	63 (28.4%)	14 (17.9%)	
BMI, n (%)			0.342
<25	86 (38.7%)	35 (44.9%)	
≥25	136 (61.3%)	43 (55.1%)	
Blood Pressure (mmHg), n (%)			0.094
Non-hypertensive	104 (46.8%)	28 (35.9%)	
Hypertensive	118 (53.2%)	50 (64.1%)	
PAD	48 (21.6%)	30 (38.5%)	0.004

Table 2 compares laboratory and biochemical parameters between TPOAb-negative and TPOAb-positive groups. However, HbA1c levels were not significantly different (P = 0.213), and a higher proportion of TPOAb-positive individuals had poor glycemic control 33 patients (42.3%) vs. 70 patients (31.5%), FT4 and FT3 were similar between groups (P = 0.509 and P = 0.088, respectively), and there was a trend toward lower FT3 in the TPOAb-positive group. Lipid profiles such as LDL-C (P = 0.305), HDL-C (P = 0.100), total cholesterol (P = 0.470), and triglycerides (P = 0.452) were not significantly different. Additionally, glucose levels were not significantly different (P = 0.578). There were no differences found in uric acid levels between the groups (P = 0.793). TPOAb positivity did not meaningfully affect glycemic control, thyroid function, lipid metabolism, and levels of uric acid overall in this cohort.

**Table 2:** Comparison of laboratory and biochemical parameters between TPOAb-negative and TPOAb-positive groups (N = 300) FT3: free triiodothyronine; FT4: free thyroxine; LDL-C: low-density lipoprotein cholesterol; HDL-C: high-density lipoprotein cholesterol; TC: total cholesterol; TG: triglycerides; HbA1C: hemoglobin A1c; TPOAb: anti-thyroid peroxidase antibody; UA: uric acid

Variable	TPOAb -ve (n = 222)	TPOAb +ve (n = 78)	P-value
HbA1c (%), n (%)			0.213
Well controlled	63 (28.4%)	20 (25.6%)	
Moderately controlled	89 (40.1%)	25 (32.1%)	
Poorly controlled	70 (31.5%)	33 (42.3%)	
FT4 (pmol/L), median (range)	14.67 (13.21-16.12)	14.17 (12.90-16.26)	0.509
FT3 (pmol/L), mean ± SD	4.58 (0.58)	4.45 (0.60)	0.088
LDL-C (mmol/L), median (range)	2.80 (2.20-3.36)	2.87 (2.31-3.47)	0.305
HDL-C (mmol/L), median (range)	1.17 (1.00-1.39)	1.11 (0.99-1.25)	0.100
TC (mmol/L), median (range)	5.01 (4.26-5.89)	5.05 (4.47-5.99)	0.470
TG (mmol/L), median (range)	1.65 (1.15-2.21)	1.69 (1.29-2.52)	0.452
Glucose (mmol/L), median (range)	7.64 (6.20-10.40)	8.46 (6.11-11.21)	0.578
UA category, n (%)			0.793
Normal	153 (68.9%)	55 (70.5%)	
Hyperuricemia	69 (31.1%)	23 (29.5%)	

A univariate binary logistic regression analysis is presented in Table 3 to examine factors related to PAD. Among the variables, TPOAb positivity was significantly associated with an increased risk of PAD (OR: 2.27, P = 0.004) and might indicate a relationship between thyroid autoimmunity and vascular complications. People with diabetes for >10 to 15 years had the greatest risk (OR 3.75; P = 0.001) for PAD development compared with those with diabetes for >15 years (OR 2.10; P = 0.045), which shows that the longer one is with the disease in the hyperglycemic state, the more it contributes to the development of PAD. Another important risk factor for PAD was poor glycemic control, and well-controlled HbA1c increased the likelihood of PAD (OR: 2.10, P = 0.043), and poorly controlled HbA1c even more (OR: 2.54, P = 0.011). On the other hand, thyroid hormone levels (FT4, FT3), lipid profiles (TC, TG, LDL-C, HDL-C), glucose levels, BMI, age, sex, and hypertension did not have a significant association with PAD risk. These findings suggest that managing thyroid autoimmunity and long-term diabetes duration is important in reducing PAD risk, as well as glycemic control.

**Table 3:** Univariate binary logistic regression analysis of factors associated with peripheral artery disease (N = 300) FT3: free triiodothyronine; FT4: free thyroxine; LDL-C: low-density lipoprotein cholesterol; HDL-C: high-density lipoprotein cholesterol; TC: total cholesterol; TG: triglycerides; HbA1C: hemoglobin A1c; TPOAb: anti-thyroid peroxidase antibody; UA: uric acid; BP: blood pressure

Variable	OR	95% CI	P-value
FT4 (pmol/L)	1.12	0.98 - 1.27	0.089
FT3 (pmol/L)	0.79	0.50 - 1.22	0.286
LDL-C (mmol/L)	0.92	0.71 - 1.20	0.543
HDL-C (mmol/L)	0.63	0.27 - 1.50	0.296
TC (mmol/L)	1.04	0.87 - 1.25	0.665
TG (mmol/L)	1.06	0.95 - 1.18	0.278
Glucose (mmol/L)	1.06	0.99 - 1.14	0.111
TPOAb-positive vs. negative	2.27	1.29 - 3.95	0.004
UA: hyperurecemia vs. normal	1.18	0.68 - 2.05	0.553
Duration: (T2DM >5–10 years vs. ≤5 years)	1.54	0.74 - 3.22	0.248
Duration: (T2DM >10-15 vs. ≤5 years)	3.75	1.68 - 8.38	0.001
Duration: (T2DM >15 vs. ≤5 years)	2.10	1.02 - 4.35	0.045
Age group: (45-64 vs. <45 years old)	0.75	0.27 - 2.08	0.578
Age group: (≥65 vs. <45 years old)	1.26	0.47 - 3.41	0.650
Sex: male vs. female	1.50	0.88 - 2.54	0.135
HbA1c moderately vs. well-controlled	2.10	1.02 – 4.31	0.043
HbA1c poorly vs. well-controlled	2.54	1.23 - 5.23	0.011
BMI category: ≥25 vs. <25	1.20	0.70 - 2.03	0.509
BP: hypertensive vs. non-hypertensive	1.10	0.65 - 1.85	0.726

**Figure 1:**
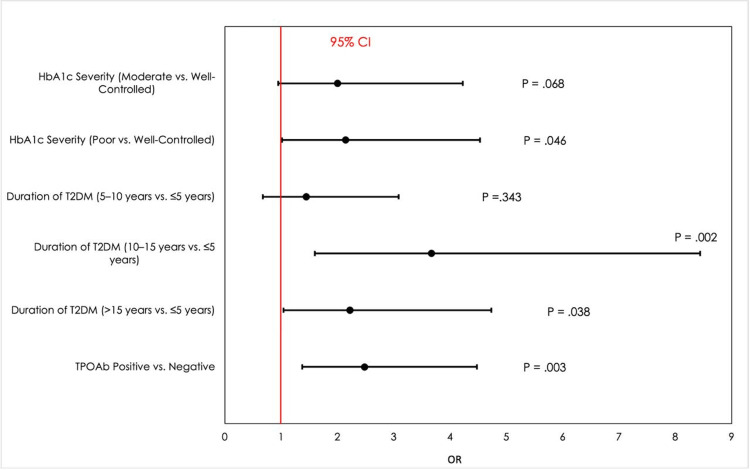
Forest plot of adjusted odds ratios for risk factors associated with peripheral artery disease in type 2 diabetes Forest plot displaying adjusted odds ratios and 95% confidence intervals for peripheral artery disease risk factors in type 2 diabetes. Significant predictors included poor hemoglobin A1c (HbA1c) control, longer diabetes duration, and anti-thyroid peroxidase antibody (TPOAb) positivity.

A multivariate binary logistic regression analysis to identify independent risk factors of PAD is presented in Table 4. Individuals with poorly controlled HbA1c had an increased risk of PAD compared to well-controlled individuals (OR: 2.14, P = 0.046), and those with moderately controlled HbA1c had a borderline significant risk (OR: 2.0, P = 0.068). In parallel, the duration of diabetes also had a significant impact; individuals who had diabetes for >10-15 years showed a significantly higher risk (OR: 3.67, P = 0.002), followed by those with diabetes for more than 15 years (OR: 2.22, P = 0.038), suggesting that prolonged hyperglycemia may have a cumulative detrimental effect on vascular health. Similarly, TPOAb positivity was independently correlated with increased odds of PAD (OR: 2.48, P = 0.003) and therefore potentially heightened the risk of macrovascular complications in association with thyroid autoimmunity. Nevertheless, PAD risk was not significantly increased by diabetes duration of >5-10 years (P = 0.343). A forest plot has been created to illustrate the relationship between HbA1c severity, diabetes duration, and TPOAb positivity for better visual presentation (Figure 1). Thus, poor glycemic control, longer diabetes duration, and TPOAb positivity prove to be key independent risk factors for PAD, while requiring careful glycemic management and further work on the role of thyroid autoimmunity in vascular disease.

**Table 4:** Multivariate binary logistic regression analysis for factors associated with peripheral artery disease (N = 300) T2DM: type 2 diabetes mellitus; HbA1C: hemoglobin A1C; TPOAb: anti-thyroid peroxidase antibody

Variable	OR	95% CI	P-value
HbA1c severity (moderate vs. well-controlled)	2.00	0.95 – 4.22	0.068
HbA1c severity (poor vs. well-controlled)	2.14	1.01 – 4.53	0.046
Duration of T2DM (>5–10 years vs. ≤5 years)	1.44	0.68 – 3.09	0.343
Duration of T2DM (>10–15 years vs. ≤5 years)	3.67	1.60 – 8.44	0.002
Duration of T2DM (>15 years vs. ≤5 years)	2.22	1.04 – 4.73	0.038
TPOAb-positive vs. negative	2.48	1.38 – 4.48	0.003

## Discussion

Several studies have reported thyroid dysfunction to be associated with T2DM [9,10]. The analysis showed a much higher proportion of females in the TPOAb-positive group compared to the TPOAb-negative group, 60 patients (76.9%) vs. 134 patients (60.4%), p = 0.008, in concordance with the predilection for female predominance of thyroid autoimmunity. Numerous studies have shown that females with T2DM have significantly more chances of having thyroid disorders (31.4% in females vs. 6.9% in males) [11]. Furthermore, PAD prevalence was greater in the TPOAb-positive group vs. the TPOAb-negative group: 30 patients (38.5%) vs. 48 patients (21.6%), p = 0.004, which could suggest a possible association between thyroid autoimmune and macrovascular complications. This finding is consistent with research that shows that thyroid autoimmunity is linked with cardiovascular diseases, including PAD [12]. Type 1 diabetes mellitus is a common chronic autoimmune disease that is known to have an elevated risk of thyroid autoimmunity [13]. However, a few studies have also shown a positive relationship with T2DM. In a study on Saudi patients with T2DM, both TPOAb and TGAb were found to be considerably more prevalent [14]. Another study also found a positive association between T2DM and TPOAb [15].

Several important factors have a significant correlation with PAD by the univariate binary logistic regression analysis. A significant relationship existed between the risks of PAD and TPOAb positivity (OR: 2.27, P = 0.004) and, therefore, between thyroid autoimmunity and vascular complications. This is consistent with previous research showing that TPOAb levels are positively associated with atherosclerosis even in the normal thyroid function range, which implies that TPOAb might be involved in endothelial remodeling and vascular complications [16]. Furthermore, the duration of diabetes was an important determinant, with the risk of PAD being highest in those with diabetes for >10 to 15 years (OR: 3.75, P = 0.001) and over 15 years (OR: 2.1, P = 0.045). This is in line with the findings of the UK Prospective Diabetes Study, which also showed that the prevalence of PAD is higher with a longer duration of diabetes [17]. Another strong risk factor was having poor control of blood sugars, in that moderately controlled HbA1c substantially raises the risk of PAD (OR: 2.1, P = 0.043) and poorly controlled HbA1c even more so (OR: 2.54, P = 0.011). This is consistent with previous studies that found a strong relation between HbA1c levels and the prevalence of PAD in diabetes patients [18]. Despite this, thyroid hormone levels (FT3, FT4), lipid parameters, glucose levels, BMI, age, sex, and hypertension were not associated with PAD risk alone. These findings underscore the need to control thyroid autoimmunity, glycemic control, and long-term diabetic duration to reduce PAD risk.

Independent risk factors for PAD, as mentioned in the multivariate binary logistic regression analysis, were several. Poorly controlled HbA1c was a significant predictor of PAD, with increased risks of PAD compared to well-controlled individuals (OR: 2.14, p = 0.046). This finding agrees with previous studies that show poor glycemic control increases limb amputation and 30-day postoperative adverse events following infra-inguinal bypass [19]. Like the previous study, duration of diabetes also played a crucial role, with diabetes duration of >10 to 15 years having the highest risk (OR: 3.67, P = 0.002) and diabetes duration of more than 15 years (OR: 2.22, P = 0.038). This is in agreement with the finding that the severity and duration of diabetes are associated with higher chances of developing lower extremity PAD [18]. In addition, TPOAb positivity was independently associated with PAD (OR: 2.48, P = 0.003), which is thyroid autoimmunity that could predispose to vascular complications. It has been shown that this dyslipidemia, a known risk factor for PAD, may be associated with subclinical and overt hypothyroidism [20].

Limitations and recommendations

This study has important implications for the relationship between TPOAb, hyperuricemia, and peripheral arterial disease in T2DM patients. Nevertheless, there are some limitations. A single-center retrospective design may prevent systemic generalizability because such variations may exist in healthcare settings, genetic predispositions, and environmental factors. In addition, the sample size was small enough to potentially reduce the statistical power of the analysis. The study has another limitation as it’s a retrospective cohort study and thus can’t give an idea about causality or progression of complications over time. Additionally, there were important confounders not included in the analysis, like lifestyle factors, use of medication, dietary habits, and inflammatory markers that may explain the observed associations. Furthermore, these findings can be improved in terms of validity by multi-center studies with bigger and more diverse populations.

## Conclusions

The findings showed that a positive TPOAb is independently associated with an increased risk of PAD, suggesting a possible role of thyroid autoimmunity in macrovascular complications. In addition, poor glycemic control and longer diabetes duration were associated with PAD, supporting the view that strict diabetes control reduces vascular complications. Future research can be done in prospective cohort studies to assess if thyroid autoimmunity and hyperuricemia have a long-term impact on PAD risk. Screening for thyroid autoantibodies may be considered in future prospective studies involving T2DM patients, particularly those with long-standing diabetes and poor glycemic control.
